# Rethinking the plasmid paradox: when plasmid costs do not affect fitness

**DOI:** 10.3389/fmicb.2026.1836467

**Published:** 2026-05-26

**Authors:** Lucy Androsiuk, Shay Tal

**Affiliations:** 1 National Center for Mariculture, Israel Oceanographic and Limnological Research Ltd., Eilat, Israel; 2 Department of Life Sciences, Ben-Gurion University of the Negev, Beer-Sheva, Israel

**Keywords:** fitness, growth-rate, microbial evolution, mobile genetic elements, plasmid ecology, plasmid paradox, rate-limiting step

## Abstract

Plasmids frequently impose measurable fitness costs on their bacterial hosts, yet they remain abundant across clinical and environmental microbiomes. This apparent contradiction, known as the plasmid paradox, has traditionally been explained through mechanisms such as horizontal gene transfer, compensatory evolution, addiction systems, and fluctuating selection. Here we suggest that part of the paradox may arise from implicit physiological assumptions embedded in most empirical measurements—specifically, the assumption that growth rate is a direct proxy for fitness and that plasmid burden necessarily reduces it. We argue that these assumptions may not hold under many ecological conditions. We formalize cell division time as the maximum of several required cellular modules, including cytoplasmic biosynthesis and membrane or envelope synthesis. If plasmid carriage primarily increases cytoplasmic demand, its cost will be expressed only when cytoplasmic processes constitute the dominant bottleneck for growth. When other modules limit division, plasmid-associated burdens may be physiologically real yet evolutionarily silent. More broadly, equating fitness with maximal exponential growth rate overlooks well-established growth-survival trade-offs in bacteria, suggesting that plasmid costs measured under optimized laboratory conditions may systematically overestimate ecological selection against plasmid carriage.

## 1 Introduction: revisiting the plasmid paradox

Plasmids often impose measurable burden on their microbial hosts. Typically, plasmids constitute around 2.5% of the total DNA content of a cell (Ramiro-Martínez et al., 2025), thereby consuming cellular resources through their maintenance and replication. Additional burden may arise from the expression of plasmid-encoded genes, the effects of plasmid-encoded proteins on microbial host physiology, and plasmid-associated processes such as conjugation or integration (Millan and MacLean, 2017).

Based on these considerations, plasmid-bearing cells would be expected to be outcompeted by plasmid-free cells unless plasmids confer compensating benefits. Conversely, if plasmids carry beneficial genes, these genes might eventually integrate into the chromosome, eliminating the need for plasmid maintenance. Together, these expectations imply that plasmids should not be evolutionarily stable. Yet plasmids are widespread and persist across virtually all environments studied (Androsiuk et al., 2023; Fogarty et al., 2024; He et al., 2026). This apparent contradiction is known as the plasmid paradox (Harrison and Brockhurst, 2012; MacLean and San Millan, 2015; Carroll and Wong, 2018; Brockhurst and Harrison, 2022).

Over the past decades, several mechanisms have been proposed to resolve this paradox. One major explanation treats plasmids as infectious genetic elements whose persistence is driven by horizontal gene transfer, allowing plasmids to spread faster than they are eliminated by selection against costly carriage (Harrison and Brockhurst, 2012). Other explanations include compensatory mutations that reduce plasmid costs, toxin-antitoxin systems that enforce plasmid maintenance, and episodic positive selection for plasmid-encoded genes (Brockhurst and Harrison, 2022).

While these mechanisms undoubtedly contribute to plasmid persistence, most of them implicitly rely on a shared assumption: that plasmid burden reduces growth rate and that reduced growth rate translates directly into reduced fitness. Implicit in this framework is the idea that cytoplasmic biosynthetic capacity, such as ribosome-driven protein synthesis and biomass accumulation, sets the pace of cell division (Scott and Hwa, 2011; Wu et al., 2022). Under such conditions, additional plasmid replication and gene expression directly reduce maximal growth rate, generating measurable selection against plasmid carriage. This assumption generally holds under nutrient-rich laboratory conditions, where exponential growth rate provides a convenient and reproducible fitness proxy (Carroll and Wong, 2018).

Here we propose that part of the plasmid paradox may arise from a tacit physiological assumption embedded in many experimental measurements: that plasmid burden necessarily affects the rate-limiting step of cell division.

## 2 Growth rate is not the sole currency of fitness

A key assumption underlying many plasmid studies is that fitness can be adequately captured by maximal exponential growth rate. Yet microbial fitness is multidimensional and often shaped by trade-offs between rapid growth and survival-related traits such as stress tolerance, repair capacity, or metabolic flexibility.

Such trade-offs are well documented in bacteria. Global regulatory programs, including the RpoS-mediated general stress response, allocate resources away from rapid growth toward enhanced stress resistance (Weber et al., 2005). In fluctuating or resource-limited environments, slower-growing organisms may exhibit higher long-term fitness because they are better prepared for environmental change.

More generally, evolutionary theory predicts that organisms maximize long-term geometric growth rate rather than instantaneous growth rate in fluctuating environments (Sæther and Engen, 2015). This perspective aligns with recent analyses highlighting the fundamental trade-offs between instantaneous exponential growth and long-term evolutionary success, demonstrating that the maximization of a single fitness component often comes at the expense of others (Bruggeman et al., 2023). Consequently, phenotypes with the highest short-term growth rate are not always the most successful in the long run.

Empirical studies illustrate this principle. For example, experiments with structured microbial populations have shown that cooperative behaviors with an apparent fitness cost can persist under spatial structure (Chuang et al., 2009). Similarly, environmental studies demonstrate that slower-growing bacterial taxa may be favored under certain conditions, such as elevated temperatures or nutrient limitation (Abreu et al., 2023). These observations highlight that fitness in microbial populations often depends on multiple traits beyond maximal growth rate.

If growth rate alone determined evolutionary success, extraordinarily fast-growing bacteria such as *Vibrio natriegens* would be expected to dominate microbial communities broadly. Instead, such extreme growth rates are typically observed only under highly permissive laboratory conditions (Weinstock et al., 2016). In natural environments, fitness often reflects a balance between proliferation and preparedness for environmental stress.

These observations suggest that equating fitness with maximal exponential growth rate may oversimplify the selective landscape experienced by bacteria in natural ecosystems.

## 3 A modular view of cell division

Another implicit assumption in many plasmid studies is that plasmid burden affects the cellular process that limits division. However, bacterial cell division is not governed by a single process. Rather, it requires the coordinated completion of multiple physiological modules, including cytoplasmic biomass accumulation, chromosome replication, envelope synthesis, and septation (Scott and Hwa, 2011; Harris and Theriot, 2016; Reyes-Lamothe and Sherratt, 2019).

If division time is determined by the slowest required process (Coltharp et al., 2016; Männik et al., 2024), it can be expressed as:

$$\tau =\max(\tau_{M},\tau_{C},\tau_{D},\ldots )$$

where each $\tau_{i}$ represents the time required to complete a necessary module. Growth rate is g=1/τ .

Let $\tau_{C}$ represent the time required to complete cytoplasmic tasks such as DNA replication and protein synthesis. If plasmid carriage primarily increases cytoplasmic load, for example by requiring additional DNA replication or plasmid-encoded protein production, it effectively increases $\tau_{C}$ by some increment δ .

Whether this increment alters overall division time depends entirely on which cellular module is rate-limiting. If cytoplasmic processes already represent the slowest step, then increasing $\tau_{C}$ directly increases τ , and plasmid costs become visible as reduced growth rate. However, if another module, such as membrane synthesis, remains slower than $\tau_{C}+\delta$ , then division time remains unchanged.

Although cellular processes are ultimately coupled through shared resources, selection acts primarily on delays that propagate to the dominant bottleneck.

**Figure 1:**
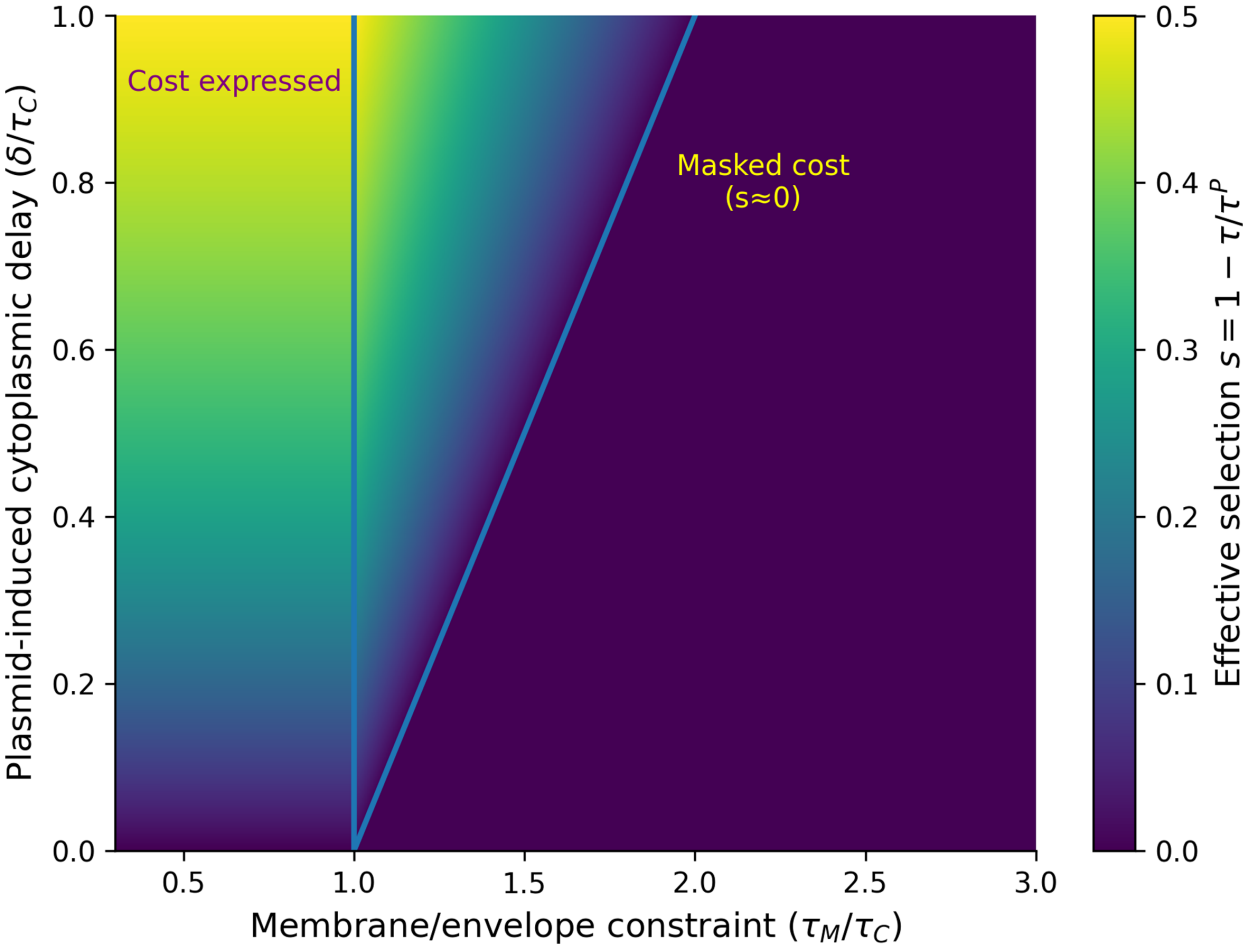
Module-specific bottlenecks can mask plasmid fitness costs. We model division time as $\tau =\max(\tau_{M},\tau_{C})$ , where $\tau_{M}$ and $\tau_{C}$ are the times required for membrane/envelope and cytoplasmic biosynthesis modules, respectively. Plasmid carriage is represented as an added cytoplasmic delay, $\tau_{C}\to \tau_{C}+\delta$ , yielding $\tau^{P}=\max(\tau_{M},\tau_{C}+\delta )$ , and an effective selection against plasmid carriage $s=1-\tau /\tau^{P}$ . The heatmap shows that when membrane/envelope processes remain rate-limiting even after δ (region right of $\tau_{M}/\tau_{C}=1+\delta /\tau_{C}$ ), plasmid costs are effectively masked ( s≈0 ), whereas costs are expressed when cytoplasmic biosynthesis is limiting.

The consequences of this architecture are illustrated in Figure 1, which maps the effective selection against plasmid carriage across regimes of module-specific limitation. The model predicts a broad region of parameter space in which plasmid burden is physiologically real yet evolutionarily silent because it does not affect the rate-limiting step.

## 4 When is cytoplasmic biosynthesis the bottleneck?

Under optimized laboratory conditions, cytoplasmic biosynthesis often limits division (Scott and Hwa, 2011; Wu et al., 2022). In such environments, $\tau =\tau_{C}$ , and increasing $\tau_{C}$ directly increases division time. Consequently, plasmid costs measured under these conditions appear as reduced exponential growth rates.

However, other physiological processes can become limiting under different environmental conditions. For example, membrane or envelope synthesis may limit cell growth when lipid availability is restricted or when envelope remodeling becomes energetically demanding (Harris and Theriot, 2016; Vadia et al., 2017; Kitahara et al., 2022). In such scenarios, $\tau_{M}$ may exceed $\tau_{C}+\delta$ . When this occurs, plasmid-induced increases in cytoplasmic demand do not alter overall division time. The plasmid burden remains physiologically present, but it no longer translates into a reduction in realized growth rate.

This perspective reframes the plasmid paradox. Rather than asking how costly genetic elements persist despite their burden, we may ask whether the measured costs are relevant to the physiological bottleneck that limits growth in natural systems.

## 5 Ecological implications: shifting bottlenecks

Environmental conditions can reshuffle which cellular process limits division. Nutrient availability, temperature, osmotic stress, oxygen limitation, and spatial structure can each shift rate limitation among cytoplasmic, envelope, and replication modules (Scott and Hwa, 2011; Harris and Theriot, 2016; Wu et al., 2022).

In structured habitats such as biofilms, sediments, and host-associated environments, diffusion constraints and envelope remodeling may impose stronger limitations than ribosomal capacity. Similarly, in fluctuating environments, lag time and stress recovery may contribute more strongly to fitness than exponential growth rate.

Recent work has shown that plasmid acquisition costs may manifest primarily as extended lag phases rather than reduced growth rates (Ahmad et al., 2023), highlighting that plasmid burdens can affect physiological dimensions not captured by simple growth-rate measurements.

If many ecological contexts impose limitations outside cytoplasmic biosynthesis, plasmid costs measured in rich media may systematically overestimate the strength of selection against plasmid carriage. Under such circumstances, plasmids need not be strongly beneficial or highly transmissible to persist; they may simply operate within physiological slack.

## 6 Testable predictions

The modular bottleneck hypothesis generates experimentally testable predictions. Shifting bottlenecks experimentally should alter the effective selection against plasmid carriage. For example, imposing envelope limitations through lipid scarcity or envelope stress could reduce measurable plasmid costs relative to cytoplasm-limited conditions.

Similarly, structured growth systems, such as biofilms or diffusion-limited chemostats, may exhibit weaker selection against plasmids than well-mixed batch cultures.

More broadly, measuring multiple components of fitness, including lag time, stress tolerance, and recovery dynamics, may reveal plasmid-associated effects that are invisible when fitness is assessed solely through exponential growth rate.

## 7 Conclusion

The plasmid paradox has traditionally been framed as a conflict between measurable fitness costs and widespread plasmid persistence. Here we suggest that part of this paradox may reflect a mismatch between how fitness is measured in the laboratory and how cell division is constrained in natural environments.

We note that the modular model presented here relies on a simplified framework where cell division is governed by discrete, independent processes and a strict single rate-limiting step. In reality, bacterial physiology is highly integrated, characterized by extensive cross-talk and synergistic effects. The translational or metabolic costs of a plasmid may simultaneously impact multiple traits, interlinking growth and survival dynamics. However, we utilize this abstracted model to demonstrate a fundamental biological possibility: that the imposition of a physiological burden does not intrinsically guarantee an equivalent reduction in realized fitness, provided that the affected module is not the dominant bottleneck in a given environment.

If plasmid burden frequently affects cellular modules that are not rate-limiting under ecological conditions, then selection against plasmid carriage may be weaker than laboratory measurements suggest. Integrating physiological bottlenecks and growth–survival trade-offs into plasmid ecology may therefore provide a more realistic framework for understanding how mobile genetic elements persist in microbial ecosystems.

## Data Availability

The original contributions presented in the study are included in the article/supplementary material, further inquiries can be directed to the corresponding author.
